# Respiratory allergy to *Blomia tropicalis*: Immune response in four syngeneic mouse strains and assessment of a low allergen-dose, short-term experimental model

**DOI:** 10.1186/1465-9921-11-51

**Published:** 2010-05-01

**Authors:** Tiana Baqueiro, Momtchilo Russo, Virgínia MG Silva, Thayna Meirelles, Pablo RS Oliveira, Eliane Gomes, Renato Barboza, Ana T Cerqueira-Lima, Camila A Figueiredo, Lain Pontes-de-Carvalho, Neuza M Alcântara-Neves

**Affiliations:** 1 Departamento de Biointeração, Instituto de Ciências da Saúde, Universidade Federal da Bahia, Av. Reitor Miguel Calmon, Sem n°. Canela, Salvador, Bahia, CEP 40110902, Brasil; 2 Núcleo de Tecnologia em Saúde, Instituto Multidisciplinar em Saúde, Universidade Federal da Bahia, Av. Olívia Flores, Candeias, Vitória da Conquista, Bahia, CEP 4503100, Brazil; 3 Departamento de Imunologia, Instituto de Ciências Biomédicas, Universidade de São Paulo; Av. Prof. Lineu Prestes, 1730, Cidade Universitária, Butantã, CEP 05508-900, Brazil; 4 Centro de Pesquisas Gonçalo Moniz, Fundação Oswaldo Cruz, Rua Waldemar Falcão, 121, Brotas, Salvador, Bahia, CEP 40296710, Brazil

## Abstract

**Background:**

The dust mite *Blomia tropicalis *is an important source of aeroallergens in tropical areas. Although a mouse model for *B. tropicalis *extract (*Bt*E)-induced asthma has been described, no study comparing different mouse strains in this asthma model has been reported. The relevance and reproducibility of experimental animal models of allergy depends on the genetic background of the animal, the molecular composition of the allergen and the experimental protocol.

**Objectives:**

This work had two objectives. The first was to study the anti-*B. tropicalis *allergic responses in different mouse strains using a short-term model of respiratory allergy to *Bt*E. This study included the comparison of the allergic responses elicited by *Bt*E with those elicited by ovalbumin in mice of the strain that responded better to *Bt*E sensitization. The second objective was to investigate whether the best responder mouse strain could be used in an experimental model of allergy employing relatively low *Bt*E doses.

**Methods:**

Groups of mice of four different syngeneic strains were sensitized subcutaneously with 100 μg of *Bt*E on days 0 and 7 and challenged four times intranasally, at days 8, 10, 12, and 14, with 10 μg of *Bt*E. A/J mice, that were the best responders to *Bt*E sensitization, were used to compare the *B. tropicalis*-specific asthma experimental model with the conventional experimental model of ovalbumin (OVA)-specific asthma. A/J mice were also sensitized with a lower dose of *Bt*E.

**Results:**

Mice of all strains had lung inflammatory-cell infiltration and increased levels of anti-*Bt*E IgE antibodies, but these responses were significantly more intense in A/J mice than in CBA/J, BALB/c or C57BL/6J mice. Immunization of A/J mice with *Bt*E induced a more intense airway eosinophil influx, higher levels of total IgE, similar airway hyperreactivity to methacholine but less intense mucous production, and lower levels of specific IgE, IgG1 and IgG2 antibodies than sensitization with OVA. Finally, immunization with a relatively low *Bt*E dose (10 μg per subcutaneous injection per mouse) was able to sensitize A/J mice, which were the best responders to high-dose *Bt*E immunization, for the development of allergy-associated immune and lung inflammatory responses.

**Conclusions:**

The described short-term model of *Bt*E-induced allergic lung disease is reproducible in different syngeneic mouse strains, and mice of the A/J strain was the most responsive to it. In addition, it was shown that OVA and *Bt*E induce quantitatively different immune responses in A/J mice and that the experimental model can be set up with low amounts of *Bt*E.

## Introduction

Exposure to house dust mite allergens is recognized as the most important risk factor for the development of allergic diseases [1-3]. Among the mites, *Dermatophagoides pteronyssinu*s and *Blomia tropicalis *are the main sources of allergens in sub-tropical and tropical regions of the world [4-6]. High frequencies of positivity to *B. tropicalis *antigens in skin prick tests have been described in asthma and rhinitis patients, such as 68.1% in Cuba [7], 91.6% in Venezuela [8], 73.3% in Taiwan [9] and 95.0% in São Paulo, Brazil [10]. There is evidence that allergens from *B. tropicalis *are distinct from, and bear only low to moderate cross-reactivity to allergens from *Dermatophagoides sp*. [11]. For instance, antibodies from allergic patients against the main *B. tropicalis *allergens (proteins of 14.3 and 27.3 kDa) do not inhibit the binding of anti-*D. pteronyssinus *antibodies to *D. pteronyssinus *antigens [4,9,11]. Thus, sensitization to *B. tropicalis *allergens is considered an independent and important cause of allergy [4,8]. These findings justify studies on species-specific diagnosis and immunotherapy for *B. tropicalis *allergy in regions where this species occurs alone or concomitantly with *D. pteronyssinus*.

Animal models that mimic the immunological and pulmonary inflammation features observed in human asthma are important tools to dissect the basic cellular and molecular mechanisms involved in the initiation and control of allergy [12]. Conventional models of allergic asthma rely on the sensitization of experimental animals to ovalbumin (OVA). However, in humans, most cases of asthma are due to aeroallergens, and OVA-induced asthma is far from being a common event. Thus, experimental asthma models using common allergens might be more relevant tools to the study of human asthma [13]. Despite the bulk of work done in humans on mite-specific allergy, data on allergic responses to *B. tropicalis *antigens in murine models are scarce [14-16]. These works were carried out using single (A/Sn or BALB/c) mouse strains, and, to the best of our knowledge, no work comparing the allergic response to *B. tropicalis *antigens in different mouse strains has been done so far. Experimental data indicate that inbred mouse strains differ in their ability to mount an allergen-induced asthmatic response [17,18]. Mice of some strains develop an intense airway hyperreactivity, eosinophilia and IgE production, while others fail to produce allergic responses [18].

The first objective of the present work was to study the murine allergic response to *B. tropicalis *using a short-term immunization protocol. The following parameters were used to measure the immune response in mice of four inbred strains (CBA/J, BALB/c, A/J and C57Bl/6): (i) the total number of leukocytes and eosinophils in the bronchoalveolar lavage fluid (BALF); (ii) the concentration of IL-4 and IL-13 cytokines and eosinophil peroxidase (EPO) in the BALF; (iii) the serum levels of anti-*B. tropicalis *IgE antibodies. *Bt*E-immunized mice of the most responsive strain (A/J strain) were then assessed for the presence of intra-bronchial mucous, airway hyperresponsiveness (AHR) to methacholine challenge and inflammatory cell infiltration in lung tissue. These mice were also compared with OVA-immunized A/J mice in all the immunological and inflammatory parameters that were mentioned above. As a second objective of the present work, mice of the best-responder strain were immunized with relatively low doses of *Bt*E aiming at obtaining a low allergen-dose, short term murine model of respiratory allergy to *B. tropicalis *that reproduced many immunological and pathological features of the human disease.

## Materials and methods

### Animals

Eight to 10 week-old CBA/J, BALB/c, A/J and C57BL/6 male mice, and 3 to 4 month-old Wistar rats, were bred and maintained at the animal houses of the Gonçalo Moniz Research Center, Oswaldo Cruz Foundation, Salvador, Brazil, and of the Biomedical Sciences Institute, University of São Paulo, São Paulo, Brazil. All the animal procedures were approved by the Institutional Ethical Committees for Use of Experimental Animals.

### *Blomia tropicalis *extract

*B. tropicalis *house dust mites were collected from bed dust in Salvador, Brazil, cloned and cultured with a powdered fish food medium (Spirulina, Alcon Gold, São Paulo, Brazil), and dry yeast (Fermipan, São Paulo, Brazil), at 25°C and 75% humidity. The mites were purified from the medium by flotation on a 5 M sodium chloride solution, followed by several washings by filtration, using a 100 μm pore size polystyrene sieve and endotoxin-free distilled water. The washings were carried out until no food residues could be seen under microscopy. The mites were lysed in 0.15 M phosphate-buffered saline, pH 7.4 (PBS), in a blender (Waring Commercial, Torrington, Connecticut, USA). Lipids from the lysate were extracted and discarded by five or six ether extractions. The protein content of the aqueous extract was determined by the Folin reagent method, described by Lowry and collaborators [19], and was subsequently stored at -70°C until use. The amount *Bt*E used in the experiments was standardized by measuring its content in *B. tropicalis *Blo t 5 allergen, measured by a commercially available capture ELISA kit (INDOOR Biotechnologies, Charlottesville, VI, USA). All used batches contained 30-40 ng of this allergen per μg of protein.

### Sensitization protocol

Groups of mice from different mouse strains were sensitized to *Bt*E by subcutaneous injections of 100 μg or 10 μg of *Bt*E adsorbed to 1.6 mg of alum [Al(OH)_3_] gel (Sigma Chemical Co., St. Louis, MO, USA) on days 0 and 7 and challenged intranasally with 10 μg of *Bt*E in 50 μl of saline on days 8, 10, 12 and 14. Four different batches of *Bt*E were used in different experiments. Control groups received only alum and were challenged with saline or with *Bt*E. In addition, groups of A/J mice were injected with 100 μg of OVA (Sigma Chemical Co., St. Louis, MO, USA) adsorbed to alum and challenged with 50 μL of saline containing 10 μg of OVA, as described above for the *Bt*E. The mice were painlessly killed 24 h after the last allergen challenge.

### Blood collection

Mice were deeply anesthetized by intraperitoneal injection with a solution containing ketamine (Ketamina Agener; União Química Farmacêutica Nacional S/A, São Paulo, Brazil) and chloral hydrate (Labsynth, São Paulo, Brazil) and blood samples from the retro orbital plexus were collected for serum antibody level determinations.

### Bronchoalveolar lavage fluid collection and cell counting

The tracheas of the dead mice were cannulated and the BALF collected in 0.5 mL of PBS containing 1% of bovine serum albumin (Sigma Chemical Co., St. Louis, MO, USA; PBS-BSA). An aliquot of the BALF cells was washed three times by centrifugation, and the cell pellet resuspended in PBS-BSA. Total cell counts were carried out using a Neubauer chamber. Differential cell counts were performed in light microscopy, according to standard morphologic criteria, by counting, in a blinded fashion, 100 cells in cytospin preparations stained with Rosenfeld's stain. Following centrifugation (400 *g*, 5 min, 4°C), supernatants of the BALF were collected and stored at -70°C for subsequent measurement of cytokine content and the pellets were used for the measurement of eosinophil peroxidase (EPO) activity.

### Eosinophil peroxidase activity in BALF

The EPO activity present in BALF was determined by means of the colorimetric assay that was described by Strath et al. [20]. Briefly, the BALF was incubated with an erythrocyte-lysing buffer, consisting of 0.15 M NH_4_Cl, 1 mM KHCO_3 _and 0.1 mM EDTA, pH 7.4, and centrifuged. The cell pellets were resuspended in PBS and lysed by three successive freezing and/thawing procedures, and then assayed for peroxidase activity in 96-well microassay plates, in duplicates, using 6.6 mM H_2_O_2 _and 1.5 mM orthophenylenodiamine (Merck, Whitehouse Station, NJ, USA).

### Cytokine assays

The BALF supernatants were stored at -70°C until used. IFN-γ and IL-4, IL-10 and IL-13 concentration measurements were assayed in commercial ELISA kits according to manufacturer's instructions (Pharmingen, St. Diego, CA, USA). Sensitivities were >5 pg/mL for IL-4, >2 pg/mL for IL-10, >0.5 pg/mL for IL-13 and >0.03 ng/mL for INF-γ.

### Lung histology

After the BALF collection, the lungs were perfused, via the heart right ventricle, to remove residual blood, immersed in 10% phosphate-buffered formalin for 24 h, followed by 70% ethanol, and embedded in paraffin. Tissues sections of 5-μm were then stained with periodic acid-Schiff (PAS) for the evaluation of mucus production. A quantitative digital morphometric analysis was performed using the application program Metamorph 6.0 (Universal Images Corp. Downingtown, PA, USA). The circunference area of the bronchi in the PAS-stained area was electronically measured and the mucus index was determined by the following formula: Mucus index = (PAS-stained area/bronquial cross-section area) × 100.

### Determination of airway responsiveness

Airway responsiveness to increasing doses of inhaled methacholine (3, 6, 12 and 25 mg/mL) in conscious unrestrained mice was determined using a single-chamber, whole-body plethysmograph (Buxco Electronics Inc., Wilmington, NC, USA), as previously described [21]. After each nebulization with methacholine, recordings were taken for 5 min. Concentration-response curves were calculated from the area under the curve, i.e. the time integral of changes in airway resistance within 20 min [22].

### ELISA for immunoglobulin isotypes

Serum anti-OVA or anti-*Bt*E IgG1 and IgG2a antibodies were measured using OVA- or *Bt*E-coated microtitre plates and biotin-conjugated anti-mouse IgG1 or anti-mouse IgG2a, respectively (Pharmingen, St. Diego, CA, USA), in conjunction with streptavidin-horseradish peroxidase, H_2_O_2 _and orthophenylenodiamine (Merck, Whitehouse Station, NJ, USA). Total IgE was detected using anti-mouse IgE-coated microtitre plates and biotin-conjugated anti-mouse IgE (UNLB Southern Biotechnology Associates, Inc., Birmingham, AL, USA), in conjunction with streptavidin-horseradish peroxidase, H_2_O_2 _and orthophenylenodiamine. The antibody concentration was obtained by interpolation into a curve obtained by concomitantly assaying different concentrations of mouse IgE.

### Passive cutaneous anaphylaxis reaction (PCA)

IgE antibody serum levels were estimated by PCA reaction, as described by Mota and Wong [23]. In brief, 0.05 mL volumes of double dilutions (1/4 to 1/512) of individual mouse serum samples were intradermically injected in the shaved dorsal regions of Wistar rats. After 48 hours, the rats received 2 mg of *Bt*E in the tail vein, diluted in 0.5 mL of saline containing 0.5 mg/mL of Evans blue (Sigma Chemical Co, St. Louis, MO, USA). The rats were painlessly killed 30 min later, and the reciprocal of the highest serum dilution to produce a blue spot with more than 5 mm of diameter was considered the PCA titer.

### Statistical analysis

The normality of the data was determined using the Komogorov-Smirnov test. In order to verify differences among more than two mouse groups, the results were analyzed using the one-way ANOVA test and the Tukey's post test. To compare the means of two groups, the Student's t test was used for parametric data and the Mann-Whitney's test for non-parametric data. All results were considered statistically significant when p ≤ 0.05.

## Results

### Cytokine, EPO and leukocyte concentrations in BALF, and IgE serum levels, in four strains of mice following sensitization and challenge with *B. tropicalis *extract

**Figure 1:**
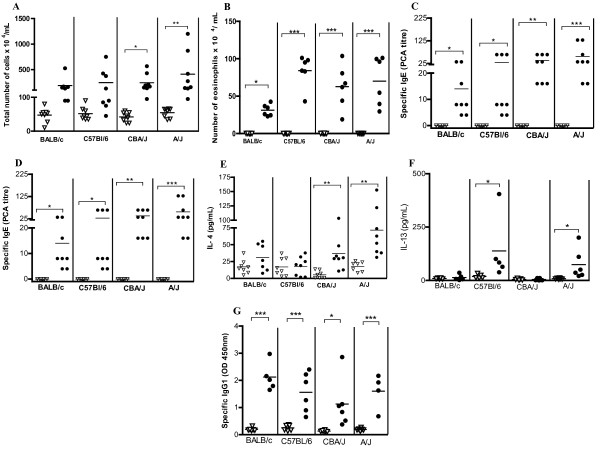
**Immune response of BALB/c, C57Bl/6, CBA/J and A/J mice sensitized with *Blomia tropicalis *extract (closed symbols) or injected with saline (open symbols)**. **(A) **Total leukocyte numbers in the bronchoalveolar lavage fluid (BALF). **(B) **Eosinophil numbers in the BALF. **(C) **Level of eosinophil peroxidase (EPO) activity in BALF. **(D) **Anti-*B. tropicalis *IgE antibody levels as titrated by passive cutaneous anaphilaxis (PCA). **(E) **IL-4 concentration in BALF. **(F) **IL-13 concentration in BALF. **(G) **Anti-*B. tropicalis *IgG1 antibody levels in blood. Each symbol corresponds to the result obtained from an individual animal. This data is representative of three independent experiments. *p < 0.05, **p < 0.01, ***P < 0.001; ANOVA and Tukey's test. P > 0.05 is not represented.

Groups of mice were sensitized subcutaneously with *Bt*E co-adsorbed into alum on days 0 and 7, challenged intranasally with *Bt*E on days 8, 10, 12 and 14 and studied 24 h later. Although the total cell counts in BALF were higher in sensitized A/J mice than in the other sensitized mouse strains, the differences were not statistically significant (p > 0.05, ANOVA test; Figure 1A). Only in A/J and CBA/J mice these total cell counts differed significantly from their saline controls (p < 0.05; Tukey's test; Figure 1A). Eosinophil numbers increased in the BALF of all sensitized mouse strains, in relation to their saline control (Figure 1B; p < 0.05 for BALB/c and p < 0.001 for A/J, CBA/J and C57Bl/6; Tukey's test). No differences in numbers of macrophage, lymphocyte and neutrophils in the BALF were observed among the mice of all four strains (p > 0.05, ANOVA; data not shown). EPO activity levels in BALF increased in all *Bt*-sensitized and challenged mice and was higher in A/J, CBA/J and C57Bl/6 mice than in BALB/c mice (p < 0.0001, p < 0.001, and p < 0.01, respectively; Tukey's test; Figure 1C). Mice from all four studied strains, sensitized and challenged with *Bt*E had higher levels of *Bt*E-specific IgE as revealed by PCA, than the alum- and saline-treated control mice (p < 0.001 for A/J, p < 0.01 for CBA/J, and p < 0.05 for C57Bl/6 and BALB/c, Tukey's test; Figure 1D). The differences in IgE titers in *Bt*E-sensitized and challenged mice in the four studied mouse strains were not statistically significant (p > 0.05; ANOVA), although A/J mice showed the highest titers, followed by the CBA/J, C57Bl/6 and BALB/c mice. IFN-γ and IL-10 concentrations in the BALF from *Bt*E-sensitized or saline-treated mice of all tested mouse strains were low, and no statistically significant differences were found among the studied groups and their negative controls (data not shown). The production of IL-4 in *Bt*E-sensitized and challenged mice was higher in A/J when compared with the other studied mouse strains (Tukey's test, p < 0.05; Figure 1E); it was followed by the production in CBA/J mice (p < 0.01, Tukey's test). *Bt*E-sensitized and challenged BALB/c or C57Bl/6 mice produced low amounts of IL-4, which were similar to those produced by their saline-treated control groups (p > 0.05, Tukey's test; Figure 1E). IL-13 production was increased in A/J and C57Bl/6 sensitized mice in comparison with the corresponding control mice (p < 0.05, Tukey's test; Figure 1F). Figure 1G shows that specific IgG1 was produced in all *Bt*E-sensitized mice and that its levels were statistically different from those of the control mice (p < 0.001 for BALB/C, C57Bl/6, and A/J mice, and p < 0.05 for CBA/J; Tukey's test).

Animals that were not subcutaneously immunized with *Bt*E (they received instead control injections of alum), and were subsequently challenged with *Bt*E, did not differ from control, non-immunized mice that were challenged with saline, in any of the studied parameters (data not shown).

### Comparison of sensitization to *Bt*E with sensitization to OVA, and presence of AHR and intra-bronchial mucus in A/J mice

**Figure 2:**
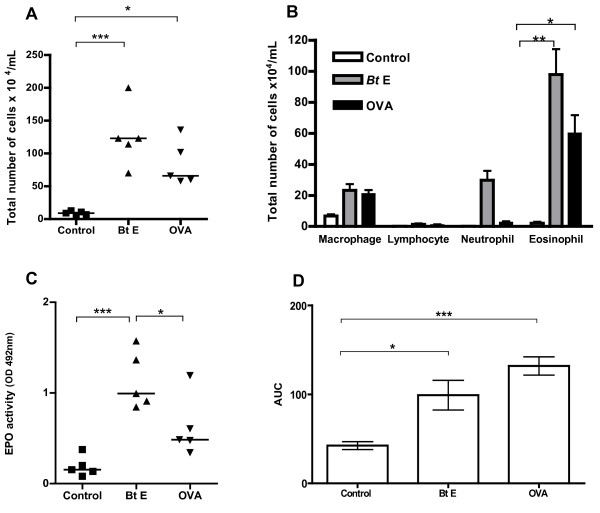
**Allergic response of A/J mice sensitized with *Blomia tropicalis *extract (Bt E) or ovalbumin (OVA) or injected with saline (Control)**. **(A) **Total leukocyte numbers in BALF. **(B) **Differential leukocyte numbers in BALF. **(C) **Level of eosinophil peroxidase (EPO) activity in BALF. **(D) **Degree of airway responsiveness, as shown by the area under the curve (AUC) of the response to methacholine × time. *p < 0.05, **p < 0.01, and ***p < 0.001 for the indicated tested groups (Tukey's test). P > 0.05 is not represented. In **A **and **C**, each symbol corresponds to the result obtained from an individual animal. In **B **and **D**, columns represent the mean result of 5 (B) or 8 (D) animals; the vertical bars represent the standard deviation of the means. Data from **A**, **B**, and **C **are representative of three experiments, and from **D **of two experiments.

**Figure 3:**
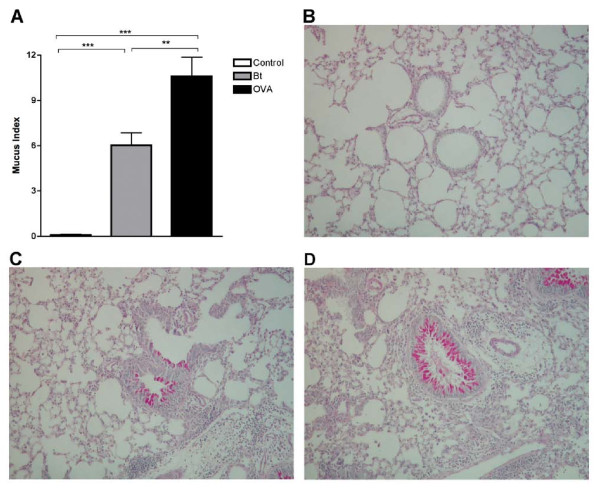
**Presence of mucus in bronchi of A/J mice sensitized with ovalbumin (OVA), *Blomia tropicalis *extract (Bt) or saline (Control)**. **(A) **Mucus Index in the bronchi. Each column represents the mean of the mucus indexes of 5 mice, and the vertical bars represent the standard deviation of the mean. **p < 0.01, ***p < 0.001, ANOVA. **(B-D) **Representative lung sections stained with periodic acid-Schiff. **(B) **saline-injected group. **(C) **OVA-sensitized group. **(D) **Bt-sensitized group. The data are representative of three independent experiments. P > 0.05 is not represented.

**Figure 4:**
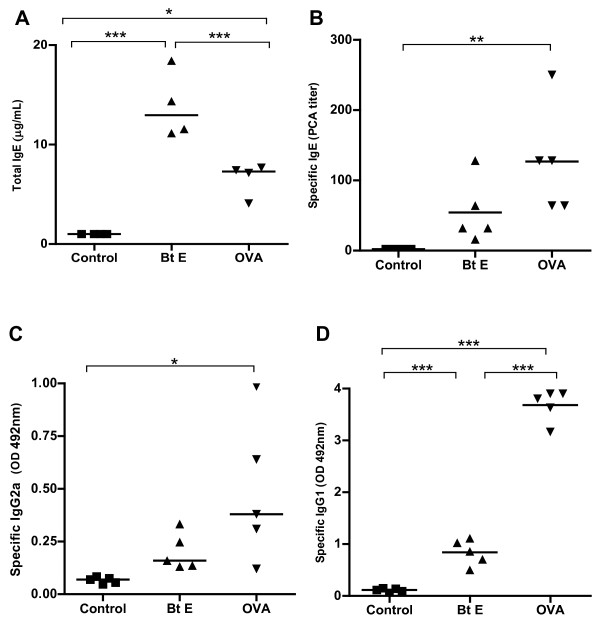
**Total IgE and specific antibody levels in the blood of A/J mice that were sensitized with ovalbumin (OVA) or *Blomia tropicalis *extract (Bt E) or injected with saline (Control)**. **(A) **Total IgE. **(B) **Anti-Bt or anti-OVA IgE antibodies. **(C) **Anti-Bt or anti-OVA IgG2a antibodies. **(D) **Anti-Bt or anti-OVA IgG1 antibodies. *p < 0.05, **p < 0.01, ***p < 0.001; Tukey's test. P > 0.05 is not represented. The data are representative of three independent experiments.

Since A/J mice had more intense allergic responses, we selected this strain to make a comparison between the *Bt*E-induced asthma model with the classical OVA-induced asthma model. Animals sensitized and challenged with *Bt*E showed higher levels of total cells and eosinophils in the BALF than control mice (p < 0.001, Figure 2A, and p < 0.01, Figure 2B; Tukey's test). OVA-sensitized mice also showed increased total cell (p < 0.05; Figure 2A; Tukey's test) and eosinophil counts (p < 0.05; Figure 2B; Tukey's test) in the BALF than the corresponding control, saline-treated animals. EPO activity in BALF was also higher in *Bt*E-sensitized than in OVA-sensitized and control mice (p < 0.05 and p < 0.001, respectively; Tukey's test; Figure 2C). Sensitization with OVA (p < 0.001, Tukey's test) and *Bt*E (p < 0.01, Tukey's test) induced AHR, as compared with control mice (Figure 2D). The mucus index was higher in mice sensitized with OVA than in mice sensitized with *Bt*E or in the mice of the saline-treated control group (Figure 3A; p < 0.001 and p < 0.01, respectively; ANOVA and Tukey's test). Representative micrographs of tissue sections of control, *Bt*E- or OVA-sensitized mice, stained with PAS, are shown respectively in Figure 3B, C and 3D. The effect of *Bt*E and OVA sensitizations on total IgE and specific antibodies levels are shown in Figure 4. Total IgE was higher in *Bt*E-sensitized animals (Figure 4A; p < 0001) and specific-IgE, IgG2a and IgG1 antibodies were higher in OVA-sensitized group (Figure 4B-D; p < 0.05, Tukey's test).

### Evaluation of a low-dose *B. tropicalis *extract protocol and lung inflammatory infiltration in A/J mice

**Figure 5:**
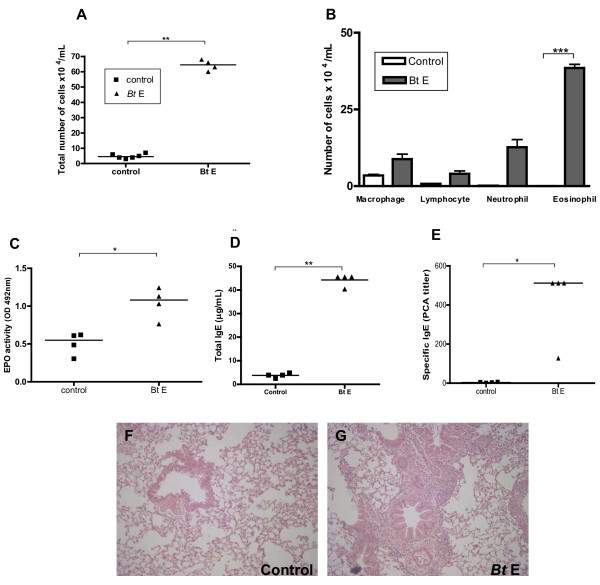
**Effect of immunization with 10 μg per injection of *B. tropicalis *extract (Bt E) on the development of experimental respiratory allergy in A/J mice**. Control mice were injected with saline (Control). (A) Total cell count in the BALF. (B) Differential cell count in the BALF. (C) Levels of eosinophil peroxidase (EPO) in the BALF. (D) total serum IgE. (E) IgE anti-Bt antibody serum titers as determined by passive cutaneous anaphylaxis (PCA). Each symbol corresponds to the result obtained from an individual animal. (F and G) representative lung sections stained with hematoxylin and eosin of a saline-injected animal (F) and a *Bt*-sensitized animal (G). The data are representative of three independent experiments. *p < 0.05, **p < 0.01, ***p < 0.001. A-D, Student's t test; E, Mann-Whitney's test. P > 0.05 is not represented.

After the observation that A/J was the best mouse strain for *Bt*E-induced asthma, we immunized these mice with a low-dose (10 μg per injection) of *Bt*E instead of the 100 μg dose per injection used in the previous experiments. A significantly larger number of cells was found in the BALF of the mice sensitized with low-dose of *Bt*E than in the BALF of the saline control group (Figure 5A; p < 0.01; Student's t test). Eosinophils were the main cellular type, followed by neutrophils, found in the BALF of mice of the *Bt*E-sensitized group, and macrophages were found in larger numbers in the saline control group (Figure 5B; p < 0.001 for differences in eosinophil counts between *Bt*E-sensitized and control group; Student's t test). The EPO activity was higher in the BALF of *Bt*E-sensitized mice than in that of negative controls (Figure 5C; p < 0.01; Student's t test). *Bt*E-sensitized animals had more total serum IgE as well as higher titers of anti-*Bt*E IgE antibodies than the saline control group (Figure 5D and 5E; p < 0.05; Student's t test and Mann-Whitney's test, respectively). The effect of sensitization and challenge with 10 μg of *Bt*E per injection on lung histology is seen in Figures 5F and 5G. *Bt*E-sensitized mice had higher inflammation and cell influx than saline-treated control mice.

## Discussion

Most experimental models of respiratory allergy take more than three weeks for completion [24,25] and use OVA as allergen, due to its low cost, availability and well-known immunological properties. However, results obtained in murine experimental models of respiratory allergy that use OVA as antigen differ from those obtained in experimental models using mite allergens. For instance, BALB/c mice respond vigorously to OVA in terms of allergic inflammation but are low responders to mite allergens [24]. Differences in allergenicity between *D. pteronyssinus *and *B. tropicalis *antigens have also being reported in experimental models of asthma [14]. In addition to allergen-dependent differences in intensity and nature of the allergic responses, the genetic makeup of the host seems to play an important role in murine models of respiratory allergy. On the other hand, a protocol developed by Eum and collaborators [26], using OVA, showed that shortening the duration of the allergic protocol did not affect the immunopathological features of the experimental disease, when it was compared with classical protocols [24]. It is described, herein, the development of a short-term protocol using *B. tropicalis *extract. The allergenity of *B. tropicalis *antigens to mice has been demonstrated before [14-16], although without a detailed investigation using different strains of mice and different doses of antigen. Using a short time model, we showed that A/J mouse strain was the best responder in terms of providing an experimental model of respiratory allergy. It responded to immunization with the highest numbers of leukocytes in the BALF, consisting mainly of eosinophils, and had high levels of EPO activity in the BALF. Additionally, there were high levels of IL-4 and IL-13 in BALF and increased levels of specific IgE in the sera. Finally, they had intense AHR. A/J mice were also considered the best responders to *Dermatophagoides sp *allergens among four studied strains [25]. Karp and collaborators [27] identified the gene encoding complement factor 5 (C5) as a susceptibility locus for allergen-induced AHR in A/J mice. This may be relevant to the human disease, as Hasegawa and collaborators have reported that polymorphism in the C3, C3a receptor, and C5 genes affect susceptibility to bronchial asthma in human beings [28].

A short-term intranasal immunization protocol with *Bt*E, by itself, consisting of two intranasal instillations of *Bt*E per week, during 3 weeks, did not lead to detectable allergic responses, indicating that the subcutaneous immunization was required to induce the respiratory allergy (data not shown). This is accordance to Takeda and collaborators' observation that the intranasal instillation alone of *Bt*E elicited an IgE antibody response only when the antigen was continuously administered for a period of over 24 weeks [15]. Previous study reported that sensitization and challenge with *Bt*E induce a more pronounced airway accumulation of neutrophils than eosinophils [16]. In our model, eosinophils were the preponderant cells in the airways, however similar numbers of neutrophils and eosinophils were found in airways when the animals were sensitized without alum (data not shown). Thus, it appears that alum is required to achieve fully polarized Th2 responses to *Bt*E.

Our data also indicate that results obtained with OVA sensitization cannot be extrapolated to other allergens. Accordingly, sensitization of A/J mice to *Bt*E led to pulmonary inflammation with eosinophil infiltrate and to total IgE increase, while OVA sensitization produced low eosinophil and IL-4 responses in this mouse strain. On the other hand, OVA sensitization led to higher mucus production, and serum levels of specific IgE, IgG1 and IgG2a than *Bt*E sensitization.

Two key mechanisms for mucus production have been identified: one activated by engagement of epidermal growth factor receptor ligands (EGFR) and the other dependent on IL-13 and STAT6 signaling [29-31]. EGFR and STAT6 signaling were not investigated in the present study, but we found increased IL-13 levels, in relation to saline-treated controls, in *Bt*E-sensitized A/J mice.

Notably, mice sensitized with *Bt*E produced higher amounts of total IgE than those sensitized with OVA, in amounts similar to those observed with immunization with helminth antigens [32]. This finding corroborated the work of Takeda and collaborators [15], who found an increase of total IgE in *Bt*E- and cholera toxin- sensitized mice. Dust mite proteins, such as Blo t 11, a paramyosin from *B. tropicalis *that is homologue to a helminth molecule, is responsible for the cross-reactivity found between helminths and dust-mite species [33], and may be leading to the non-specific IgE stimulation in *Bt*E-sensitized mice found in this and in the above mentioned work. Another hypothesis is that proteases present in the *Bt*E cleave CD23, a negative regulator of IgE production [34].

Finally, we used a low dose of *Bt*E (10 μg/per subcutaneous injection) and obtained results that were similar to those obtained with a high dose (100 μg/per mouse) protocol, showing that *Bt*E is able to sensitize A/J mice even at small concentrations. This model may constitute a better approximation to a natural allergenic sensitization, in which allergic individuals tend to be exposed to low allergen doses, independently of the entry route, than the so far published experimental murine models, that use higher *Bt*E doses [[14-16] and [35]].

## Conclusions

Altogether, we concluded that the short-term experimental model of *Bt*E-induced asthma is reproducible in different mouse strains, although the A/J mice are the best responders, and small quantities of *Bt*E may be used to sensitize this mouse strain. We also concluded that a murine experimental model of respiratory allergy that uses *Bt*E as allergen differs quantitatively in immunological and pathological parameters when compared with the classical experimental model that uses OVA as allergen.

## Abbreviations

AHR: Airway hyperresponsiveness; BALF: Bronchoalveolar lavage fluid; *Bt*E: *Blomia tropicalis *extract; EPO: Eosinophil peroxidase; IFN-γ: Interferon gamma; IgE: Imunoglobulin E; IgG: Imunoglobulin G; IL-4: Interleukin 4; IL-10: Interleukin 10; IL-13: Interleukin 13; OVA: Ovalbumin; PBS: 0.15M phosphate-buffered saline, pH 7.4; PBS/BSA: PBS containing 1% of bovine serum albumin; PCA: Passive cutaneous anaphylaxis.

## Competing interests

The authors declare that they have no competing interests.

## Authors' contributions

TB conducted the majority of the experiments involving different mouse strains, the OVA × *Bt*E comparison experiments and wrote the first manuscript draft. MR contributed in planning the experiments and reviewing the manuscript. VMGS, TM and PRSO helped in the experiments on different mouse strains. EG and RB helped in the experiments with OVA- and *Bt*E-induced asthma models. ATC L and CAF carried out the experiments on low dose of *Bt*E. 6. LPC participated in planning the experiments and reviewing the manuscript. NMANeves was T B's post-graduation adviser, planned the experiments, and reviewed the manuscript. All authors read and approved the final manuscript.
